# Umbrella review of basket trials testing a drug in tumors with actionable genetic biomarkers

**DOI:** 10.1186/s12885-022-10421-w

**Published:** 2023-01-13

**Authors:** Alyson Haslam, Timothée Olivier, Jordan Tuia, Vinay Prasad

**Affiliations:** 1 University of California San Francisco, 550 16th St, 2nd Fl, CA 94158 San Francisco, USA; 2 Department of Epidemiology and Biostatistics, UCSF Mission Bay Campus | Mission Hall: Global Health & Clinical Sciences Building | 550 16th St, 2nd Fl, CA 94158 San Francisco, USA; 3 Department of Oncology, Geneva University Hospital, 4 Gabrielle-Perret-Gentil St, 1205 Geneva, Switzerland

**Keywords:** Basket trials, Genetic biomarker, Response rate

## Abstract

**Background:**

The utilization of basket trials in oncology has gained popularity because of the drive for precision medicine and the increasing ease of genetically profiling tumors. However, it is unknown if this has translated into patient benefit, either through higher response rates because of precision treatment or because of increasing options for less-common tumor types that are less represented in oncology drug trials. We sought to characterize basket studies for oncology drugs targeting a genetic biomarker, determine the responses for various tumor types and genetic biomarkers, and test for correlation between the number of participants in each tumor basket and the incidence of the respective tumor.

**Methods:**

We conducted a retrospective cross-sectional review of oncology basket trials on Embase or clinicaltrials.gov with published data. We included studies that reported on oncology drugs that target a genetic biomarker. We examined the response for basket trial participants, stratified by tumor type and genetic biomarker and the correlation between the number of participants in each tumor basket and the incidence of the respective tumor.

**Results:**

The overall response rate for all 25 included trials was 23%. The response for each genetic biomarker ranged from 0 to 69%, and for half of the genetic biomarkers, the response rate ranged from 0 to 100%, depending on tumor type. There is low correlation between the number of participants in each tumor basket and the incidence of the respective tumor (66.41 + -0.20x, R^2^ = 0.003, *p* = 0.75).

**Conclusion:**

While there has been an increase in the number of published basket trials and individuals included in these trials, the response rate is low, but varies widely, depending on tumor type and genetic biomarker.

**Supplementary Information:**

The online version contains supplementary material available at 10.1186/s12885-022-10421-w.

## Background

Basket trials, in contrast to traditional studies that enroll patients with the same tumor type, often test a drug that targets a specific molecular alteration, regardless of the underlying primary tumor. Basket trials have gained attention in recent years because of the efficiency in testing a drug in multiple tumor types in a single trial. An advantage of these types of trials is that less common tumor types with fewer treatment options can be tested because of the lower number of people needed for testing, and drugs can be tested in a shorter, more efficient trial [1]. Further, the possibility of a treatment option makes trial participation desirable for patients with these types of cancers [2]. The success of basket trials lies in not only being able to identify a molecular marker for the cancer, but also to identify a drug that successfully targets the particular molecular marker and having the molecular marker present in a wide range of tumor types [3]. Additionally, success of a drug may be further affected by patient selection based on a tumor’s sub-variants that result in a heterogeneity of response [4].

The ability to comprehensively test a genome profile instead of testing for a single biomarker has fueled the enthusiasm for precision medicine, including treatment and testing [4]. As a result, the number of basket trials protocols published increased from 2 in 2009 to 67 in 2019, with 92% of them testing a drug in patients with cancer [5].

Several systematic reviews of basket trials have previously been published [5–8]. These have provided instructive information on the general landscape of published and unpublished basket trials in the medical literature at-large, [5] as well as a more focused view in oncology [7]. These have helped to enumerate the number of trials that have begun, dates of trial activities, where the trials are being conducted, and biomarkers being targeted. Another review of basket trials provides responses, by tumor type, for basket trials that used biomarker eligibility criterion for the basis of enrolment, but this review is older and includes results for only a handful of basket trials that have published results [6]. Finally, one review has been more focused in providing an estimation of the risks and benefits of basket trials testing immunotherapy therapies only [6].

These recent reviews have provided limited estimates of the benefit of drugs tested in basket trials, by reporting response at a broad level (e.g., tumor type) at best, and lack information on the benefit by molecular target. We therefore sought to conduct an updated umbrella review of basket trials in oncology to determine tumor types and genetic biomarkers being studied and to enumerate the response to the drugs, overall and by tumor type and biomarker.

## Methods

### Literature *search*

We sought to assemble a list of oncology basket trials testing an anti-cancer drug. Specifically, we were interested in (1) trials that did not restrict tumor type, (2) used a genomic biomarker for trial enrolment inclusion criteria, (3) administered an anti-cancer drug as part of the intervention, and (4) reported response rates by individual tumor type for all treated tumor types. If there was no published study report for a trial meeting these inclusion criteria, it was excluded from the analytic sample.

We systematically searched Embase for all publications on basket trials using the terms “basket AND ‘clinical trial’/de” and searched for all articles published through our search date (March 16, 2022). We also searched (January 3, 2022) for all basket trials on clinicaltrials.gov by using the terms “basket” and filtering by “interventional trial”. We also searched for basket trials discussed in review articles that came up in our search. We defined basket trials as those that tested a drug in multiple tumor types. Our search and abstraction methods are based on previously used methods [7].

Using the trial identification number, we removed any duplicates and searched for trial information on clinicaltrials.gov or other trial registration website. We abstracted trial-level information, including the year the study began, drug name, genomic biomarker used for participant inclusion, tumor types, phase, intervention model (randomized, single arm, etc.), estimated enrollment, and trial group name (if one was listed).

Using the trial identifiers, we searched for published trials reporting on the efficacy of the drug (i.e., response rates). In many cases, this information was provided in articles identified through the Embase search, but other articles were identified through publications listed on the trial registration website. If we could not find published response rates by using these two methods, we searched Google Scholar using the trial identifier or trial group name (i.e., search results identified through either of these identifiers). We abstracted the median age, the percentage of participants who were male/female, the total number of participants included in the analysis, and the number of people with a response rate (complete and partial) for each tumor type and overall.

For ease of data presentation, we combined similar cancer histologies (e.g., sarcomas were combined into a single category). These combinations are reported in the Supplemental Methods. Trials including tumors based on microsatellite instability (MSI) or deficiency in DNA mismatch repair (MMR) were categorised as a single biomarker (MSI/MMR).

We used data from the American Cancer Society (2022) to determine US incidence (per 100,000) for cancers, where available. The incidences of less-common cancers were estimated from literature reports (Supplemental Table 1), including converting incident case counts from national data sources (e.g., SEER) to incidence rates. We also looked for the frequency (%) of tumor genetic biomarkers for all cancers by searching the published literature (Supplemental Table 2). If multiple frequencies were reported, we used the frequency from the source using the most number of people/samples.

For each drug tested in identified trials, we searched US Food and Drug Administration (FDA) drug labels for approval indications and dates for tumors with a tested genetic biomarker to see whether the tested drug had received approval for the tested indication.

#### Statistical analysis

We calculated frequencies and percentages of responders and non-responders for each tumor type. We further calculated number of individuals included for each biomarker type. We also calculated a correlation coefficient and coefficient of determination from an unadjusted linear regression for the number of participants in each tumor basket and the incidence of the respective tumor. We created plots of response rates for each tumor basket, by genetic biomarker and number of participants for each tumor basket by incidence (per 100,000) of the given tumor. We conducted all analyses using R software, version 3.6.2 and Microsoft Excel. In accordance with 45 CFR § 46.102(f), this study was not submitted for institutional review board approval because it involved publicly available data and did not involve individual patient data.

## Results

There were 547 records identified from Embase and Clinicaltrials.gov. We removed 354 records during the title screen and 69 duplicate records. We then excluded reviewed studies because they did not restrict trial eligibility based on biomarker status (*n* = 80), there were no published responses for the trial (*n* = 44), or did not report tumor-specific responses (*n* = 29). Supplemental Fig. 1 shows the process for study selection and the reasons trials were included or excluded.

We found 25 basket studies in oncology that restricted study enrollment to participants with a genetic biomarker and reported efficacy results (Table 1). Two studies reported separate results for more than one genetic marker, for a total of 29 drugs/biomarkers. The median number of participants for the studies was 48 (IQR: 30–122), and the median age was 60 (IQR: 58–62). The median percentage of participants who were female was 59 (IQR: 51–68), and the median percentage of participants who were male was 41 (IQR: 32–49).

**Table 1:** Characteristics of oncology basket studies that restrict on genomic biomarker (*N* = 25 studies and 1966 participants)

	Trials, median (IQR) or n (%)	Participants, n (%)
Total number of participants	48 (30–122)	1966 (100)
Age	60 (58–62)	
Percent male (*n* = 24 studies)	41 (32–49)	
Males, number (*n* = 24 studies)		651 (36.9)
Percent female (*n* = 24 studies)	59 (51–68)	
Females, number (*n* = 24 studies)		1113 (63.1)
Response rate %	23.1 (8–30)	
Progression-free survival, months (*n* = 16 studies)	4 (3–5)	
Overall survival, months (*n* = 13 studies)	10 (6–14)	
Phase (*n* = 24 studies)		
I	1 (4.0)	22 (1.1)
II	23 (92.0)	1758 (89.4)
Not indicated	1 (4.0)	186 (9.5)
Intervention model (*n* = 24 studies)		
Randomized	1 (4.0)	129 (6.5)
Single arm	16 (64.0)	929 (36.7)
Parallel, not randomized	7 (28.0)	722 (40.6)
Not indicated	1 (4.0)	186 (9.4)
Year		
2001	1 (4.0)	186 (9.5)
2010	1 (4.0)	298 (15.2)
2012	1 (4.0)	122 (6.2)
2013	5 (20.0)	430 (21.9)
2014	5 (20.0)	468 (23.8)
2015	5 (20.0)	85 (4.3)
2016	5 (20.0)	235 (12.0)
2017	1 (4.0)	13 (0.7)
2018	1 (4.0)	129 (6.6)
Biomarker (*N* = 1839 evaluable patients participants)		
AKT		35 (1.9)
ALK		4 (0.2)
BRAF		246 (14.1)
BRCA 1/2		298 (16.2)
EGFR		9 (4.8)
FGFR		48 (2.6)
HER2		292 (15.9)
HER3		16 (0.9)
Hedgehog		21 (1.1)
KIT, PDGFRA, or PDGFRB		170 (9.2)
KRAS		129 (7.0)
MMR/MSI		128 (7.0)
NRAS		47 (2.6)
NTRK		109 (5.9)
PI3K		165 (9.0)
ROS1		4 (0.2)
RTK		80 (4.4)
TSC1, TSC2, or MTOR		30 (1.6)
TSC1, TSC2, NF1, NF2 or STK11		8 (0.4)

In all 25 trials, there were 1966 participants – 651 (36.9%) males and 1113 (63.1%) females. Twenty-two (1.1%) participants were included in phase I trials, and 1758 participants (89.4%) were included in phases II trials. Most participants were included in trials with either a single arm intervention model (*n* = 929; 36.7%) or parallel, non-randomized study (*n* = 722; 40.6%).

There were 41 tumor types, represented in the 25 trials (Supplemental Fig. 2). Ovarian cancer (*n* = 260), colorectal cancer (*n* = 245), non-small cell lung cancer (*n* = 223), and sarcoma (*n* = 155) were the most represented tumor types. Response was evaluated in 1839 participants.

The overall response rate for all included trials was 23%. Of trials with more than 10 people, salivary (52%), thyroid (47%), hypereosinophilic syndrome (43%), and prostate (40%) were the conditions with the highest response rate.

**Fig. 1:**
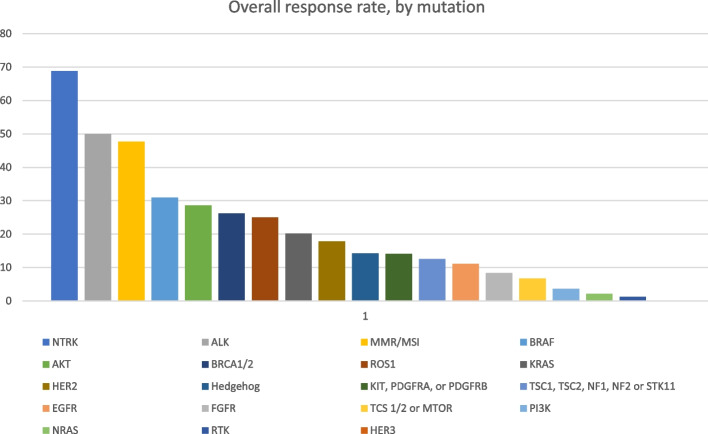
Overall response rate, by genetic biomarker for basket studies testing an oncology drug in oncology basket trials focusing on a genomic biomarker

**Fig. 2:**
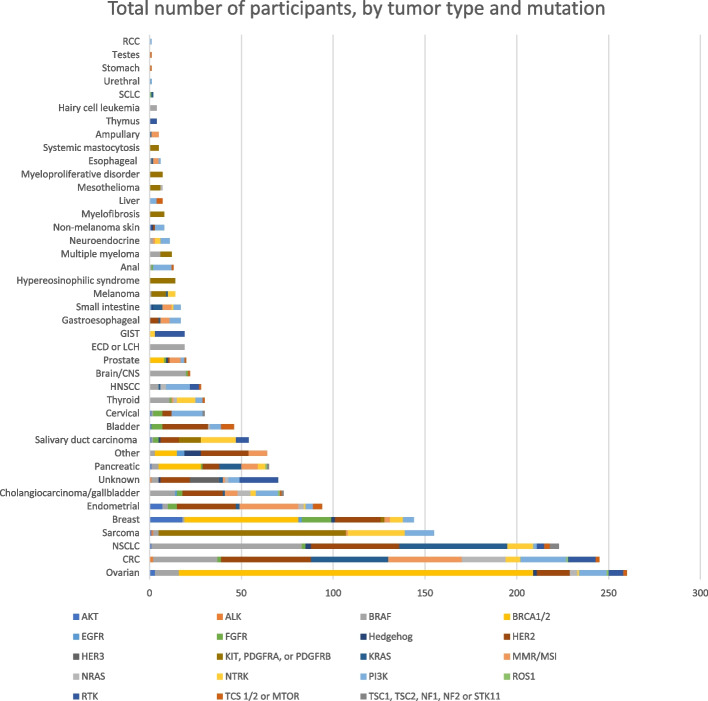
Total number of participants, by tumor type and biomarker for basket trials for oncology drugs tested in oncology basket trials focusing on a genomic biomarker

There were 19 genetic biomarker categories represented in the trials (Fig. 1). NTRK mutations had the highest response rate (69%), followed by ALK (50%), MMR/MSI (48%), and BRAF (31%) mutations/alterations. Figure 2 shows the number of participants by tumor type and genetic biomarkers.

**Fig. 3:**
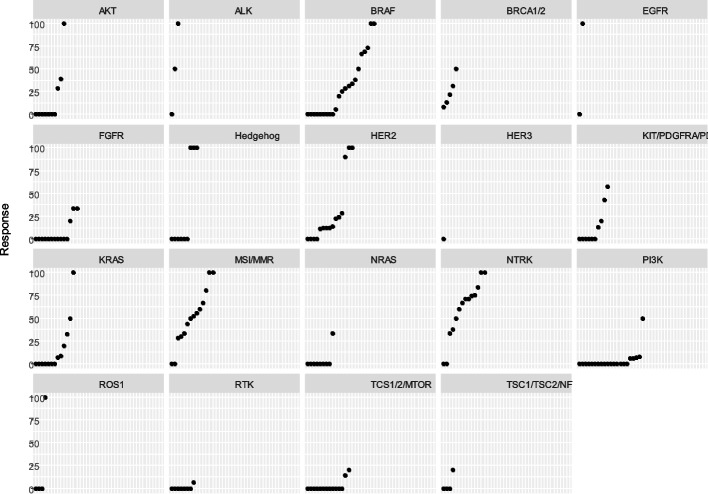
Response rates for the different tumor types, by genomic biomarker in oncology basket trials

Figure 3 shows the response rate for the different tumor types, by genomic biomarker, in oncology basket trials. For half of the biomarkers (AKT, ALK, BRAF, EGFR, Hedgehog, HER2, KRAS, MSI, NTRK, and ROS1), depending on the tumor type, the response rate ranged from 0 to 100%.

The correlation between the number of participants in each tumor basket and the incidence of the respective tumor was − 0.05, indicating low correlation (66.41 + -0.20x, R^2^ = 0.003, *p* = 0.75). If excluding non-melanoma skin tumor types, the correlation was 0.38 (7.64 + 0.15x, R^2^ = 0.14; *p* = 0.02). Supplemental Fig. 3 shows the scatterplot of the correlation between the number of participants in each tumor basket and the incidence of the respective tumor type. Ovarian, colorectal, and lung cancers over-represented tumor types. The Supplemental Fig. 4 shows the correlation between the number of participants in each tumor basket and the percentage of tumors with a given biomarker. The genetic biomarkers that are overrepresented in basket trials are BRCA1/2 mutations, HER2 overexpression, and BRAF mutations.

To date, 12 (of 29 drugs/biomarkers tested; 41.4%) studies reported on drugs that have never been FDA approved for the specific indication; nine (31.0%) studies reported on drugs that eventually received approval for the genetic biomarker in a single tumor type; 5 (17.2%) studies reported on drugs that received approval for the genetic biomarker in multiple tumor types; and 3 (10.3%) studies reported on drugs that had already been approved for the tested genetic indication in a single tumor type.

## Discussion

Our results provide updated results on basket trials that include a genetic biomarker, [8] with the addition of 17 trials and seven genetic biomarkers categories. The response rate is essentially unchanged and remains low.

While the median response rate was relatively low at 23%, some biomarkers, such as NTRK, MSI, and ALK, had better overall response than others. Larotrectinib and entrectinib were both tested and FDA approved for tumors with NTRK mutations, but the frequency of this mutation is about 0.28%, and appears to occur more frequently in rarer cancers than in more common cancers [9]. ALK mutations are more common (~ 2.8%), [10] but most cancers with this mutation are non-small cell lung cancer, melanoma, and colorectal, which are more common tumor types.

We found that response rates for a given genetic biomarker had wide variation, depending on tumor type, with some biomarkers having a zero response in some tumor types, yet 100% response in other tumor types. This suggests that broadly targeting a genetic biomarker, regardless of tumor type, reduces the overall effectiveness. This is important to note because, to date, there have been six drugs approved for tumor agnostic indications, which are based on the results of basket trials, with varied responses. In considering these biomarker-targeting drugs for approval, individual tumor response should also be considered, in addition to response rate for all tumors combined.

A recognized advantage of basket trials is that it allows researchers to study drugs in rare cancers, yet we found that the tumor types most represented in these trials were some of the most common tumor types in the general population (e.g., ovarian, colorectal, and non-small cell lung cancer). Further, few of the drugs tested in basket trials that later received FDA approval were approved for a rare cancer type (vemurafenib for ECD, larotrectinib and entrectinib for NTRK tumors, dabrafenib plus trametinib for thyroid, and imatinib for hypereosinophilic syndrome and myelodysplastic syndrome), whereas the majority were for more common tumor types (breast, lung, melanoma, and colorectal). These findings suggest that there needs to be a greater effort in recruiting patients with rare tumor types in order to more fully benefit from these types of trials.

A limitation of basket trials is that the same molecular alteration may not have the same impact on all tumor types. BRAF inhibition is one example, among others. For example, the efficacy of vemurafenib in targeting BRAF V600E mutation in patients with melanoma, [11] was not reproducible in colon cancers [12]. This variability in response has prompted the development and implementation of newer statistical methods for determining efficacy, in an effort to reduce false-positives because of multiple baskets [13].

Accordingly, a limitation of basket trials is that overall results may be driven by some tumor types, allowing the drug to be prescribed in all tumor types with limited data based on tumor-agnostic approvals. Larotrectinib was the second tissue-agnostic FDA approval for adult and pediatric patients with solid tumors and NTRK alterations. In trials leading to the approval of larotrectinib, salivary gland tumors (22%) and soft tissue sarcoma (20%) were overrepresented [14]. These same questions were raised with the KEYNOTE-158 trial, [15] leading to the approval of pembrolizumab for tumors with TMB > 10 mut/Mb, regardless their origin. As we have previously commented, no patients with prostate cancer were included in this trial, whereas 5% of them met the biomarker threshold and could be treated based on the tumor-agnostic approval, with no data [16].

### Strengths and limitations

There are strengths and limitations. First, this updated analysis provides the most comprehensive umbrella review of modern biomarker-specific oncology basket trials with reported results, to our knowledge. Second, we explored an original research question by comparing tumor types included in basket trials with population-based incidence.

There are also limitations to our analysis. First, because we only included basket trials that authors specifically identified as basket trials, our collection of trials may not be complete. However, our search was systematic, we used few restrictions in our search, and we reviewed other review articles to help us better identify the most possible trials. Second, the incidence of each type of cancer is likely overestimated because we used the higher estimates when a range was provided and some cancer types, especially the less-common tumor types, could have been counted twice (specific and broad tumor categories). Third, we only included trials if there were published data on them. Consequently, our results do not apply to all molecularly guided basket trials conducted, which could overestimate the benefit of these types of therapies in our analysis, because of publication bias toward publishing the most favorable results. Fourth, because the use of these drugs is mostly limited to those with metastatic cancers, our results are not generalizable to all patients with cancer.

## Conclusion

In conclusion, we found that while there has been an increase in the number of published basket trials and individuals included in these trials, little progress has been made on increasing the number of participants with rare tumor types, and response rates vary widely, depending on tumor type and genetic biomarker. Approvals for drugs targeting a genetic biomarker should evaluate response by tumor and not just an overall response rate.

## Supplementary Information

**Additional file 1: Supplementary Material 1**.

**Additional file 2: Supplementary Material 2**.

## Data Availability

The datasets used and/or analyzed during the current study available from the corresponding author on reasonable request.

## Abbreviations

FDA: Food and Drug Administration
