# Supplementary material for: Umbrella review of basket trials testing a drug in tumors with actionable genetic biomarkers
Source: BMC Cancer. 2023 Jan 13;23:46. doi: 10.1186/s12885-022-10421-w (PMC9840247; doi:10.1186/s12885-022-10421-w)
Supplement: Supplementary file 1 — Additional file 1: Supplementary Material 1. [file 12885_2022_10421_MOESM1_ESM.docx]

Supplemental Methods

We combined several tumor types to make studies more comparable and for ease of data presentation. Biliary, cholangiocarcinoma, and gallbladder were combined; bladder and urothelial were combined; endometrial included adenocarcinoma (serous and endometrioid), leiomyosarcoma of the uterus, and uterine; gastroesophageal (GE) included the GE junction; head and neck included the ameloblastoma of mandible, cystic carcinoma of maxillary sinus, and trachea; sarcomas included those that were soft-tissue, infantile fibrosarcoma, myxofibrosarcoma, synovial sarcoma, dermatofibrosarcoma protuberans, leiomyosarcoma, liposarcoma, chondrosarcoma, Ewing's sarcoma, angiosarcoma, rhabdomyosarcoma, aggressive fibromatosis, chordoma, desmoplastic small round cell tumor, neurofibrosarcoma, malignant schwannoma, and osteosarcoma; vulvar and vaginal were combined with cervical; brain and central nervous system included astrocytoma, glioblastoma of corpus callosum, astrocytoma of optic nerve, astroblastoma of temporal lobe, xanthoastrocytoma of parietal lobe, sarcoma of parietal occipital lobes, anaplastic ependymoma, and oligodendroglioma/ganglioglioma; Erdheim-Chester disease and Langerhans cell histiocytosis were combined; and appendix was combined into small intestine. Unless otherwise specified, lung cancers were listed as non-small cell lung cancer.

Supplemental Table 1. Tumor types and response rates for oncology basket trial studies

|  | Total | Incidence | Incidence rate (per 100,000) |
| --- | --- | --- | --- |
| Lung | 225 | 236740 | 52[^1^](https://sciwheel.com/work/citation?ids=13191063&pre=&suf=&sa=0&dbf=0) |
| CRC | 245 | 151030 | 37.7[^1^](https://sciwheel.com/work/citation?ids=13191063&pre=&suf=&sa=0&dbf=0) |
| Sarcoma | 155 | 13190 | 3.4[^1^](https://sciwheel.com/work/citation?ids=13191063&pre=&suf=&sa=0&dbf=0) |
| Breast | 144 | 290560 | 128.3[^1^](https://sciwheel.com/work/citation?ids=13191063&pre=&suf=&sa=0&dbf=0) |
| Endometrial | 94 | 65950 | 27.8[^1^](https://sciwheel.com/work/citation?ids=13191063&pre=&suf=&sa=0&dbf=0) |
| Liver and intrahepatic bile duct | 80 | 41260 | 9.5[^1^](https://sciwheel.com/work/citation?ids=13191063&pre=&suf=&sa=0&dbf=0) |
| Ovarian | 260 | 19880 | 10.6[^1^](https://sciwheel.com/work/citation?ids=13191063&pre=&suf=&sa=0&dbf=0) |
| Unknown | 70 | 30620 | 9.2[^1^](https://sciwheel.com/work/citation?ids=13191063&pre=&suf=&sa=0&dbf=0) |
| Salivary | 54 | 2380 | 1[^2^](https://sciwheel.com/work/citation?ids=13191029&pre=&suf=&sa=0&dbf=0) |
| Bladder | 46 | 81180 | 18.7[^1^](https://sciwheel.com/work/citation?ids=13191063&pre=&suf=&sa=0&dbf=0) |
| Pancreatic | 65 | 62210 | 13.3[^1^](https://sciwheel.com/work/citation?ids=13191063&pre=&suf=&sa=0&dbf=0) |
| Thyroid | 30 | 43800 | 14.6[^1^](https://sciwheel.com/work/citation?ids=13191063&pre=&suf=&sa=0&dbf=0) |
| HNSCC | 28 | 54000 | 11.5 |
| ECD or LCH | 19 | 1700 | 0.55[^3,4^](https://sciwheel.com/work/citation?ids=13191034,13191035&pre=&pre=&suf=&suf=&sa=0,0&dbf=0&dbf=0) |
| GIST | 19 | 6000 | 0.7[^1^](https://sciwheel.com/work/citation?ids=13191063&pre=&suf=&sa=0&dbf=0) |
| Small intestine | 17 | 11790 | 2.5[^1^](https://sciwheel.com/work/citation?ids=13191063&pre=&suf=&sa=0&dbf=0) |
| Gastroesophageal | 17 | 47020 | 7.1[^1^](https://sciwheel.com/work/citation?ids=13191063&pre=&suf=&sa=0&dbf=0) |
| Ampullary | 5 |  | 0.59[^5^](https://sciwheel.com/work/citation?ids=13191038&pre=&suf=&sa=0&dbf=0) |
| Brain/CNS | 22 | 20050 | 6.3[^1^](https://sciwheel.com/work/citation?ids=13191063&pre=&suf=&sa=0&dbf=0) |
| Melanoma | 14 | 99780 | 21.5[^1^](https://sciwheel.com/work/citation?ids=13191063&pre=&suf=&sa=0&dbf=0) |
| Hypereosinophilic syndrome | 14 | 26 | 0.036[^6^](https://sciwheel.com/work/citation?ids=3459916&pre=&suf=&sa=0&dbf=0) |
| Cervical/vulvar/vaginal | 30 | 20430 | 8.4[^1^](https://sciwheel.com/work/citation?ids=13191063&pre=&suf=&sa=0&dbf=0) |
| Multiple myeloma | 12 | 34470 | 7.1[^1^](https://sciwheel.com/work/citation?ids=13191063&pre=&suf=&sa=0&dbf=0) |
| Prostate | 20 | 268490 | 112.17[^1^](https://sciwheel.com/work/citation?ids=13191063&pre=&suf=&sa=0&dbf=0) |
| Neuroendocrine | 11 | 12000 | 2.89[^7^](https://sciwheel.com/work/citation?ids=13191043&pre=&suf=&sa=0&dbf=0) |
| Anal | 13 | 9440 | 1.9[^1^](https://sciwheel.com/work/citation?ids=13191063&pre=&suf=&sa=0&dbf=0) |
| Thymus | 4 | 400 | 0.15[^8^](https://sciwheel.com/work/citation?ids=13191045&pre=&suf=&sa=0&dbf=0) |
| Myelofibrosis | 8 | 4986 | 1.5[^9^](https://sciwheel.com/work/citation?ids=13191050&pre=&suf=&sa=0&dbf=0) |
| Myeloproliferative disorder | 7 | 7990 | 1.4[^10^](https://sciwheel.com/work/citation?ids=13191051&pre=&suf=&sa=0&dbf=0) |
| Mesothelioma | 7 | 3000 | 0.74[^11^](https://sciwheel.com/work/citation?ids=13191053&pre=&suf=&sa=0&dbf=0) |
| Systemic mastocytosis | 5 | 7226 | 0.46[^12^](https://sciwheel.com/work/citation?ids=13191055&pre=&suf=&sa=0&dbf=0) |
| Hairy cell leukemia | 4 | 800 | 0.62[^13^](https://sciwheel.com/work/citation?ids=13191056&pre=&suf=&sa=0&dbf=0) |
| Esophageal | 6 | 20640 | 4.2[^1^](https://sciwheel.com/work/citation?ids=13191063&pre=&suf=&sa=0&dbf=0) |
| Urethral | 1 | 60 | 4.3[^14^](https://sciwheel.com/work/citation?ids=13191058&pre=&suf=&sa=0&dbf=0) |
| Stomach | 1 | 26380 | 2.9[^1^](https://sciwheel.com/work/citation?ids=13191063&pre=&suf=&sa=0&dbf=0) |
| Testes | 1 | 9910 | 5.9[^1^](https://sciwheel.com/work/citation?ids=13191063&pre=&suf=&sa=0&dbf=0) |
| RCC | 1 | 79000 | 17.3[^1^](https://sciwheel.com/work/citation?ids=13191063&pre=&suf=&sa=0&dbf=0) |
| Non-melanoma skin | 8 | 5400000 | 1624[^15^](https://sciwheel.com/work/citation?ids=13191060&pre=&suf=&sa=0&dbf=0) |

Supplemental Table 2. Included trial information and frequency of mutation

| NCT number | Year | Drug | Target | Number of participants evaluable for response | Frequency of mutation (%) |
| --- | --- | --- | --- | --- | --- |
| NCT04439344 | 2016 | Binimetinib | NRAS | 47 | 2.9[^16^](https://sciwheel.com/work/citation?ids=8769156&pre=&suf=&sa=0&dbf=0) |
| NCT01524978 | 2012 | Vemurafenib | BRAF V600 | 122 | 5.0[^17^](https://sciwheel.com/work/citation?ids=13191075&pre=&suf=&sa=0&dbf=0) |
| NCT01831726 | 2013 | Dovitinib | RTK | 80 | 21.9[^18^](https://sciwheel.com/work/citation?ids=11720354&pre=&suf=&sa=0&dbf=0) |
| NCT01833169 | 2013 | Bbuparlisib | PI3K | 146 | 13.0[^19^](https://sciwheel.com/work/citation?ids=6211618&pre=&suf=&sa=0&dbf=0) |
| NCT01953926 | 2013 | Neratinib | HER2 | 125 | 3.5[^20^](https://sciwheel.com/work/citation?ids=5953661&pre=&suf=&sa=0&dbf=0) |
|  |  |  | HER3 | 16 | 2.9[^21^](https://sciwheel.com/work/citation?ids=13191091&pre=&suf=&sa=0&dbf=0) |
| NCT02091141 | 2014 | Pertuzumab | HER2 | 151 | 3.5[^20^](https://sciwheel.com/work/citation?ids=5953661&pre=&suf=&sa=0&dbf=0) |
|  |  | Erlotinib | EGFR | 49 | 2.8[^22^](https://sciwheel.com/work/citation?ids=13191081&pre=&suf=&sa=0&dbf=0) |
|  |  | Vemurafenib | BRAF | 21 | 5.0[^17^](https://sciwheel.com/work/citation?ids=13191075&pre=&suf=&sa=0&dbf=0) |
|  |  | Vismodegib | Hedgehog pathway | 9 | 0.7[^23^](https://sciwheel.com/work/citation?ids=12505993&pre=&suf=&sa=0&dbf=0) |
| NCT02201212 | 2014 | Everolimus | TSC1 or TSC2 mutations or activating MTOR mutations | 30 | 7.9[^24^](https://sciwheel.com/work/citation?ids=3800886&pre=&suf=&sa=0&dbf=0) |
| NCT02304809 | 2014 | Vemurafenib | BRAF | 78 | 5.0[^17^](https://sciwheel.com/work/citation?ids=13191075&pre=&suf=&sa=0&dbf=0) |
| NCT02352844 | 2015 | Everolimus | TSC1, TSC2, or MTOR | 12 | 7.9[^24^](https://sciwheel.com/work/citation?ids=3800886&pre=&suf=&sa=0&dbf=0) |
| NCT02407509 | 2013 | RO5126766 | BRAF | 22 | 5.0[^17^](https://sciwheel.com/work/citation?ids=13191075&pre=&suf=&sa=0&dbf=0) |
| NCT02568267 | 2014 | ENTRECTINIB | NTRK | 54 | 0.3[^25^](https://sciwheel.com/work/citation?ids=6419021&pre=&suf=&sa=0&dbf=0) |
| NCT02576431 | 2014 | Larotrectinib | NTRK | 55 | 0.3[^25^](https://sciwheel.com/work/citation?ids=6419021&pre=&suf=&sa=0&dbf=0) |
| NCT02675829 | 2016 | Ado-trastuzumab emtansine | HER2 | 58 | 3.5[^20^](https://sciwheel.com/work/citation?ids=5953661&pre=&suf=&sa=0&dbf=0) |
| NCT03017521 | 2017 | TAS-117 | PI3K | 13 | 13.0[^19^](https://sciwheel.com/work/citation?ids=6211618&pre=&suf=&sa=0&dbf=0) |
| NCT03600883 | 2018 | Sotorasib | KRAS | 129 | 11.0[^23^](https://sciwheel.com/work/citation?ids=12505993&pre=&suf=&sa=0&dbf=0) |
| NCT04439123 | 2016 | Capivasertib | AKT | 35 | 1.0[^19^](https://sciwheel.com/work/citation?ids=6211618&pre=&suf=&sa=0&dbf=0) |
| NCT04439214 | 2016 | Nivolumab | MMR/MSI | 47 | 7.0[^26^](https://sciwheel.com/work/citation?ids=13191095&pre=&suf=&sa=0&dbf=0) |
| NCT04439240 | 2016 | AZD4547 | FGFR | 48 | 7.1[^27^](https://sciwheel.com/work/citation?ids=5579797&pre=&suf=&sa=0&dbf=0) |
| NCT04439253 | 2015 | Crizotinib | ROS1 | 4 | 4.0[^28^](https://sciwheel.com/work/citation?ids=13191104&pre=&suf=&sa=0&dbf=0) |
| NCT04439266 | 2015 | Crizotinib | ALK | 4 | 2.8[^29^](https://sciwheel.com/work/citation?ids=13191070&pre=&suf=&sa=0&dbf=0) |
| NCT04439292 | 2015 | Dabrafenib + trametinib | BRAF | 35 | 5.0[^17^](https://sciwheel.com/work/citation?ids=13191075&pre=&suf=&sa=0&dbf=0) |
| NCT02465060 | 2015 | Copanlisib | PI3K | 30 | 13.0[^19^](https://sciwheel.com/work/citation?ids=6211618&pre=&suf=&sa=0&dbf=0) |
| Not indicated | 2001 | Imatinib | KIT, PDGFRA, or PDGFRB | 186 | 6.4[^18^](https://sciwheel.com/work/citation?ids=11720354&pre=&suf=&sa=0&dbf=0) |
| NCT01078662 | 2010 | Olaparib | BRCA1/2 | 298 | 0.3[^30^](https://sciwheel.com/work/citation?ids=10848322&pre=&suf=&sa=0&dbf=0) |
| NCT01876511 | 2013 | Nivolumab | MMR/MSI | 86 | 3.8[^31^](https://sciwheel.com/work/citation?ids=4801482&pre=&suf=&sa=0&dbf=0) |

[**References**](https://sciwheel.com/work/bibliography)

[1.    National Institute of Health. Cancer Stat Facts. Surveillance, Epidemiology, and End Results. Accessed June 20, 2022. https://seer.cancer.gov/statfacts/](https://sciwheel.com/work/bibliography/13191063)

[2.    Markman M. Salivary gland cancer. May 19, 2022. Accessed June 20, 2022. https://www.cancercenter.com/cancer-types/head-and-neck-cancer/types/salivary-gland-cancer](https://sciwheel.com/work/bibliography/13191029)

[3.    Shea CR. Langerhans Cell Histiocytosis. Medscape. June 12, 2020. Accessed June 20, 2022. https://emedicine.medscape.com/article/1100579-overview#:~:text=Frequency,reported%20in%20the%20United%20States](https://sciwheel.com/work/bibliography/13191034)

[4.    FDA Grants Priority Review and Breakthrough Therapy Designation for Zelboraf (vemurafenib) in Erdheim-Chester Disease with BRAF V600 Mutation. Genentech. August 7, 2017. Accessed June 20, 2022. https://www.gene.com/media/press-releases/14676/2017-08-07/fda-grants-priority-review-and-breakthro](https://sciwheel.com/work/bibliography/13191035)

[5.    Ramai D, Ofosu A, Singh J, John F, Reddy M, Adler DG. Demographics, tumor characteristics, treatment, and clinical outcomes of patients with ampullary cancer: a Surveillance, Epidemiology, and End Results (SEER) cohort study. *Minerva Gastroenterol Dietol*. 2019;65(2):85-90. doi:10.23736/S1121-421X.18.02543-6](https://sciwheel.com/work/bibliography/13191038)

[6.    Crane MM, Chang CM, Kobayashi MG, Weller PF. Incidence of myeloproliferative hypereosinophilic syndrome in the United States and an estimate of all hypereosinophilic syndrome incidence. *J Allergy Clin Immunol*. 2010;126(1):179-181. doi:10.1016/j.jaci.2010.03.035](https://sciwheel.com/work/bibliography/3459916)

[7.    Neuroendocrine Tumors: Statistics. Cancer.Net. Accessed June 20, 2022. https://www.cancer.net/cancer-types/neuroendocrine-tumors/statistics](https://sciwheel.com/work/bibliography/13191043)

[8.    Key Statistics About Thymus Cancers. American Cancer Society. Accessed June 20, 2022. https://www.cancer.org/cancer/thymus-cancer/about/key-statistics.html#:~:text=Although%20thymic%20tumors%20are%20the,each%20year%20is%20not%20known).](https://sciwheel.com/work/bibliography/13191045)

[9.    Tefferi A. Primary Myelofibrosis. NORD. Accessed June 20, 2022. https://rarediseases.org/rare-diseases/primary-myelofibrosis/](https://sciwheel.com/work/bibliography/13191050)

[10.   Mesa RA, Mehta J, Wang H, et al. Epidemiology of Myeloproliferative Disorders in US - a Real World Analysis. *Blood*. 2012;120(21):2834-2834. doi:10.1182/blood.V120.21.2834.2834](https://sciwheel.com/work/bibliography/13191051)

[11.   Key Statistics About Malignant Mesothelioma. American Cancer Society. Accessed June 20, 2022. https://www.cancer.org/cancer/malignant-mesothelioma/about/key-statistics.html#:~:text=Mesothelioma%20is%20fairly%20rare%20in,cases%20are%20diagnosed%20each%20year.](https://sciwheel.com/work/bibliography/13191053)

[12.   Bista A, Uprety D, Vallatharasu Y, et al. Systemic mastocytosis in united states: A population based study. *Blood*. 2018;132(Supplement 1):1830-1830. doi:10.1182/blood-2018-99-120192](https://sciwheel.com/work/bibliography/13191055)

[13.   Munoz J. Hairy Cell Leukemia. NORD. Accessed June 20, 2022. https://rarediseases.org/rare-diseases/hairy-cell-leukemia/#:~:text=Hairy%20cell%20leukemia%20affects%20about,to%2080%20years%20of%20age.](https://sciwheel.com/work/bibliography/13191056)

[14.   Aleksic I, Rais-Bahrami S, Daugherty M, Agarwal PK, Vourganti S, Bratslavsky G. Primary urethral carcinoma: A Surveillance, Epidemiology, and End Results data analysis identifying predictors of cancer-specific survival. *Urol Ann*. 2018;10(2):170-174. doi:10.4103/UA.UA_136_17](https://sciwheel.com/work/bibliography/13191058)

[15.   Skin Cancer (Non-Melanoma): Statistics. Cancer.Net. Accessed June 20, 2022. https://www.cancer.net/cancer-types/skin-cancer-non-melanoma/statistics](https://sciwheel.com/work/bibliography/13191060)

[16.   Prior IA, Hood FE, Hartley JL. The frequency of ras mutations in cancer. *Cancer Res*. 2020;80(14):2969-2974. doi:10.1158/0008-5472.CAN-19-3682](https://sciwheel.com/work/bibliography/8769156)

[17.   Gatalica Z, Burnett K, Bender R, Feldman R, Vranic S, Reddy S. 213 BRAF mutations are potentially targetable alterations in a wide variety of solid cancers. *Eur J Cancer*. 2015;51:S31. doi:10.1016/S0959-8049(16)30101-0](https://sciwheel.com/work/bibliography/13191075)

[18.   Hechtman JF. NTRK insights: best practices for pathologists. *Mod Pathol*. 2022;35(3):298-305. doi:10.1038/s41379-021-00913-8](https://sciwheel.com/work/bibliography/11720354)

[19.   Millis SZ, Ikeda S, Reddy S, Gatalica Z, Kurzrock R. Landscape of Phosphatidylinositol-3-Kinase Pathway Alterations Across 19 784 Diverse Solid Tumors. *JAMA Oncol*. 2016;2(12):1565-1573. doi:10.1001/jamaoncol.2016.0891](https://sciwheel.com/work/bibliography/6211618)

[20.   Pahuja KB, Nguyen TT, Jaiswal BS, et al. Actionable activating oncogenic ERBB2/HER2 transmembrane and juxtamembrane domain mutations. *Cancer Cell*. 2018;34(5):792-806.e5. doi:10.1016/j.ccell.2018.09.010](https://sciwheel.com/work/bibliography/5953661)

[21.   Verlingue L, Hollebecque A, Lacroix L, et al. Human epidermal receptor family inhibitors in patients with ERBB3 mutated cancers: Entering the back door. *Eur J Cancer*. 2018;92:1-10. doi:10.1016/j.ejca.2017.12.020](https://sciwheel.com/work/bibliography/13191091)

[22.   Liu H, Zhang B, Sun Z. Spectrum of EGFR aberrations and potential clinical implications: insights from integrative pan-cancer analysis. *Cancer Commun (Lond)*. 2020;40(1):43-59. doi:10.1002/cac2.12005](https://sciwheel.com/work/bibliography/13191081)

[23.   Mendiratta G, Ke E, Aziz M, Liarakos D, Tong M, Stites EC. Cancer gene mutation frequencies for the U.S. population. *Nat Commun*. 2021;12(1):5961. doi:10.1038/s41467-021-26213-y](https://sciwheel.com/work/bibliography/12505993)

[24.   Zhang Y, Kwok-Shing Ng P, Kucherlapati M, et al. A Pan-Cancer Proteogenomic Atlas of PI3K/AKT/mTOR Pathway Alterations. *Cancer Cell*. 2017;31(6):820-832.e3. doi:10.1016/j.ccell.2017.04.013](https://sciwheel.com/work/bibliography/3800886)

[25.   Okamura R, Boichard A, Kato S, Sicklick JK, Bazhenova L, Kurzrock R. Analysis of NTRK Alterations in Pan-Cancer Adult and Pediatric Malignancies: Implications for NTRK-Targeted Therapeutics. *JCO Precis Oncol*. 2018;2018. doi:10.1200/PO.18.00183](https://sciwheel.com/work/bibliography/6419021)

[26.   Liu Y, Chen L, Zhang S, et al. Somatic mutations in genes associated with mismatch repair predict survival in patients with metastatic cancer receiving immune checkpoint inhibitors. *Oncol Lett*. 2020;20(4):27. doi:10.3892/ol.2020.11888](https://sciwheel.com/work/bibliography/13191095)

[27.   Helsten T, Elkin S, Arthur E, Tomson BN, Carter J, Kurzrock R. The FGFR Landscape in Cancer: Analysis of 4,853 Tumors by Next-Generation Sequencing. *Clin Cancer Res*. 2016;22(1):259-267. doi:10.1158/1078-0432.CCR-14-3212](https://sciwheel.com/work/bibliography/5579797)

[28.   ROS1. My Cancer Genome: Genetically Informed Cancer Medicine. Accessed June 20, 2022. https://www.mycancergenome.org/content/gene/ros1/](https://sciwheel.com/work/bibliography/13191104)

[29.   ALK Mutation. My Cancer Genome; Genetically Informed Cacner Medicine. Accessed June 20, 2022. https://www.mycancergenome.org/content/alteration/alk-mutation/](https://sciwheel.com/work/bibliography/13191070)

[30.   Maxwell KN, Domchek SM, Nathanson KL, Robson ME. Population frequency of germline BRCA1/2 mutations. *J Clin Oncol*. 2016;34(34):4183-4185. doi:10.1200/JCO.2016.67.0554](https://sciwheel.com/work/bibliography/10848322)

[31.    Le DT, Durham JN, Smith KN, Wang H, Bartlett BR, Aulakh LK, Lu S, Kemberling H, Wilt C, Luber BS, Wong F. Mismatch repair deficiency predicts response of solid tumors to PD-1 blockade. *Science.* 2017 Jul 28;357(6349):409-13.](https://sciwheel.com/work/bibliography/4801482)
