# Supplementary material for: Umbrella review of basket trials testing a drug in tumors with actionable genetic biomarkers
Source: BMC Cancer. 2023 Jan 13;23:46. doi: 10.1186/s12885-022-10421-w (PMC9840247; doi:10.1186/s12885-022-10421-w)

Supplemental Figure 1. PRISMA flowchart of selection process for basket studies testing drugs in patients with a molecular biomarker.

**Identification of studies**

Records identified from:

Embase (n = 321)

Clinicaltrials.gov (n = 226)

Records excluded in title screen

Embase (n = 209)

Clinicaltrials.gov (n = 145)

Full record screened:

Embase (n = 112)

Clinicaltrials.gov (n = 81)

Review articles (n = 54)

Duplicated records excluded

(n = 69)

**Screening**

Trials excluded (124):

Not restricted by biomarker (n = 80)

No published results (n = 44)

Trials assessed for eligibility

(n = 178)

Trials excluded:

Reported results for selected tumors (n = 29)

Trials assessed for response reporting (n = 54)

Trials included in review

(n = 25)

**Included**

*From:*  Page MJ, McKenzie JE, Bossuyt PM, Boutron I, Hoffmann TC, Mulrow CD, et al. The PRISMA 2020 statement: an updated guideline for reporting systematic reviews. BMJ 2021;372:n71. doi: 10.1136/bmj.n71

Supplemental Figure 2. Number of responders and non-responders in basket trials for oncology drugs tested in a tumor with a genetic biomarker and response rate for each tumor type.


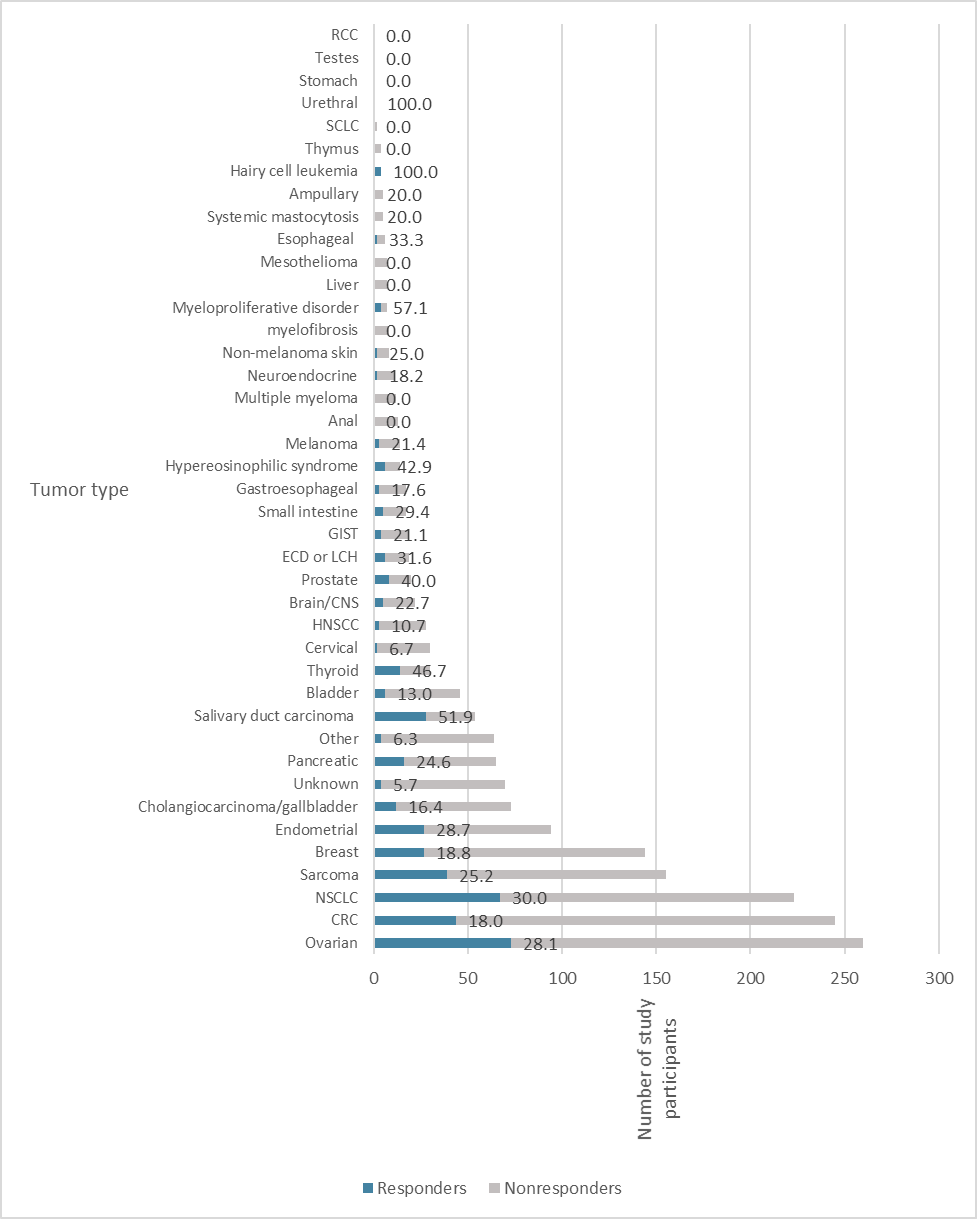


Supplemental Figure 3. Scatterplot and correlation between the number of participants in each basket and the US incidence rate of cancers represented in oncology basket trials restricting by genomic biomarker. The blue line is the regression line.


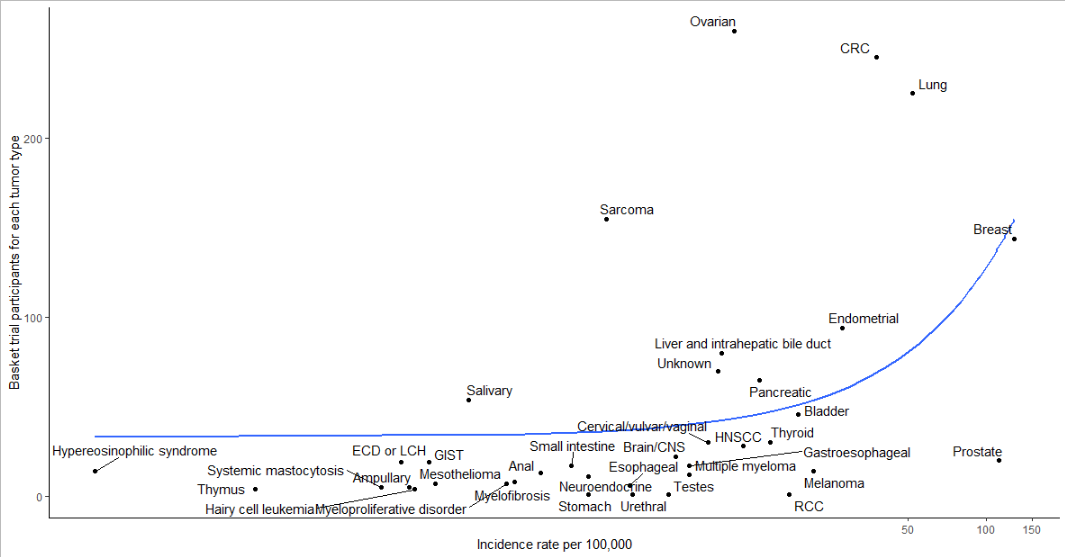


Supplemental Figure 4. Plot of the number of basket trial participants for each genetic biomarker, by the percentage of tumor types with a given biomarker.


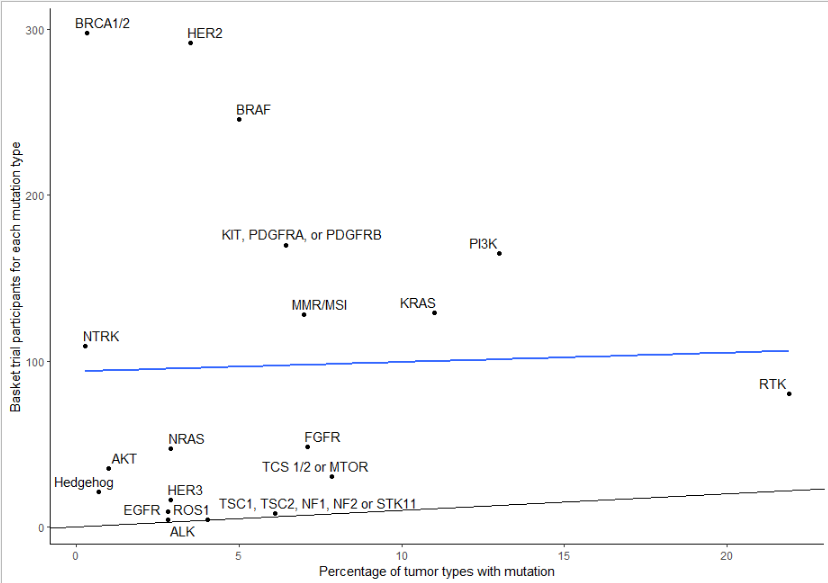

Supplement: Supplementary file 2 — Additional file 2: Supplementary Material 2. [file 12885_2022_10421_MOESM2_ESM.docx]
